# Dynamics in the expression of programmed death ligand 1 and cluster of differentiation 163 in the tumor microenvironment of uterine cervical cancer: a single-center retrospective study

**DOI:** 10.1186/s13014-023-02230-7

**Published:** 2023-02-23

**Authors:** Yusaku Miyata, Etsuyo Ogo, Toshi Abe, Hideki Hirata, Naotake Tsuda, Kimio Ushijima, Akihiko Kawahara, Jun Akiba, Hitoshi Obara, Tatsuyuki Kakuma

**Affiliations:** 1 Department of Radiology, School of Medicine, Kurume University, 67 Asahimachi, Kurume, Fukuoka 830-0011 Japan; 2 Department of Radiotherapy, St. Mary’s Hospital, 422 Tsubukuhonmachi, Kurume, Fukuoka 830-8543 Japan; 3 Department of Obstetrics and Gynecology, School of Medicine, Kurume University, 67 Asahimachi, Kurume, Fukuoka 830-0011 Japan; 4 Department of Diagnostic Pathology, Kurume University Hospital, 67 Asahimachi, Kurume, Fukuoka 830-0011 Japan; 5 Biostatistics Center, Kurume University, 67 Asahimachi, Kurume, Fukuoka 830-0011 Japan

**Keywords:** Uterine cervical cancer, External beam radiotherapy, Brachytherapy, Programmed death ligand 1, Cluster of differentiation 163, Tumor microenvironment

## Abstract

**Background:**

Radiotherapy (RT) destroys cancer cells and activates the immune system while suppressing the immunity of tumor-associated tissues, including the tumor microenvironment (TME). However, to date, no anti-tumor therapeutic strategy that uses these immune mechanisms has been established. This study investigated changes in the immunity of the TME during standard radical RT for cervical cancer combined with external beam RT and brachytherapy and determined whether these changes affect prognosis.

**Methods:**

Twenty-six patients who had completed radical RT for cervical cancer were categorized into the following two groups according to whether the cancer recurred and/or metastasized within 2 years after the start of treatment: treatment failure (n = 14) and treatment success (n = 12). We assessed the expression of programmed death 1, programmed death ligand 1 (PD-L1), cluster of differentiation (CD) 8, CD68, CD163, Forkhead box protein P3, and hypoxia-inducible factor-1α in the TME of cervical tissues collected periodically during treatment and evaluated the difference in expression rates of each marker between the success and failure groups and assessed its effect on prognosis.

**Results:**

The expression levels of PD-L1 and CD163 in the TME in the treatment success group were lower than those in the treatment failure group at the midpoint during brachytherapy (*p* < 0.01 and *p* = 0.08, respectively), and the 2-year progression-free-survival (PFS) rate depended on the expression levels of PD-L1 and CD163 (*p* = 0.04 and *p* = 0.02, respectively).

**Conclusions:**

The expression rates of CD163 and PD-L1 in the TME during brachytherapy were related to treatment response and the 2-year PFS. This study may increase our understanding of tumor-associated immunity in the TME and aid in the development of therapies targeting PD-L1 or M2 macrophages in the TME in conjunction with RT, especially brachytherapy, for cervical cancer patients.

## Background

External beam radiotherapy (EBRT) and brachytherapy with or without chemotherapy are effective definitive treatments for uterine cervical cancer, and a good prognosis is somewhat assured [1]. However, we sometimes experience cases of early recurrence and metastasis after treatment. A reason for the limited therapeutic response is that cervical cancer cells evade anti-tumor immunity. For example, in squamous cell carcinoma of the cervix, human papillomavirus (HPV) 16 is integrated into the programmed death ligand 1 (PD-L1) locus, leading to increased expression of PD-L1 [2]. Expressed PD-L1 on the surface of HPV-infected tumor cells and/or other multiple tissues, including hematopoietic cells, and programmed death 1 (PD-1) on the surface of T cells inhibit the anti-tumor effect of a cluster of differentiation (CD) 8 + T cells (cytotoxic T cells) [3–5]. Therefore, it is crucial for the successful treatment of cervical cancer to regulate tumor-suppressive immune responses.

Radiotherapy (RT) destroys cancer cells, activates the immune response, and induces immunogenic cell death. When T cells are restored from the suppressive state by PD-1/PD-L1 inhibitors, highly immunogenic neoantigens emerging from RT are recognized, and anticancer immunity is triggered. Some studies have suggested that patients with tumors overexpressing PD-L1 have improved clinical outcomes with anti-PD-1 directed therapy [6]. Additionally, several clinical trials have been conducted using anti-PD-1/PD-L1 antibodies in combination with RT, including KEYNOTE-158 (NCT02628067) [7] and CheckMate 358 (NCT02488759) [8], have used anti-PD-1/PD-L1 antibodies in combination with RT. In contrast, recent studies have shown that RT suppresses immunity against tissues surrounding the tumor, known as the tumor microenvironment (TME) [9]. When TME acquires an immunosuppressive character, it becomes resistant to RT. Furthermore, RT induces DNA double-strand breaks (DSBs) that are lethal to cancer cells, while PD-L1 expression is upregulated during the DSB repair process [10].

Definitive RT for cervical cancer comprises two radiation delivery methods, including EBRT and brachytherapy, with different doses per fraction and delivery schedules, resulting in non-uniform treatment intensity for the tumor and surrounding tissues. However, to the best of our knowledge, little is known about immune changes in TME induced by different RT techniques and dose schedules. Therefore, this study aimed to retrospectively investigate changes in the tumor-associated immune system caused by RT in TME during treatment and analyze whether these changes affect the prognosis.

## Methods

### Patients

Patients with uterine cervical cancer who were pathologically diagnosed and underwent radical RT with EBRT and high-dose-rate intracavity brachytherapy (HDR-ICBT) in our hospital between May 2012 and March 2019 were retrospectively examined, and 30 patients, whose cervical tissue samples had been collected before, during (weekly), and after treatment for assessment of treatment efficacy, were enrolled in this study. One patient who did not undergo HDR-ICBT was excluded from this population, and three others were excluded because their specimens were not collected routinely during treatment. Overall, 26 patients were finally included in this study. We obtained approval from the ethics committee of our institution to use these cervical tissue samples for this study (No. 19191). Because of this study’s exploratory and preliminary nature, sample size calculations or power analyses were not conducted.

### Treatment and follow-up

The whole (n = 25) or small pelvis (n = 1) was irradiated with EBRT with 0°, 90°, 180°, and 270° portals, using high-energy 10 MV X-ray photons from a linear accelerator at a daily fraction of 1.8 to 2.0 Gy. A midline block was administered after pelvic field irradiation of 19.8–39.8 Gy. Overall, approximately 50 Gy (49.6–50.4 Gy) was administered, with an additional boost of 6–10 Gy in 3–5 fractions for intrapelvic lymph node metastases. If there were metastases in the para-aortic lymphatic chains, they were also included in the irradiation field. The small pelvic irradiation field was designed based on the report of Ohara et al. [11] without a midline block. Furthermore, HDR-ICBT was applied with a 2D plan using a Co-60 source before or after administering a midline block. The HDR treatment plan was calculated using HDRplus™ (Eckert & Ziegler BEBIG GmbH, Berlin, Germany). A total dose of 20–40 Gy in 4–8 fractions (median, 30 Gy in 6 fractions) was prescribed for Point A in the Manchester system. The International Federation of Gynecology and Obstetrics [12] patients with stage IB2 and IIA2-IVA underwent concurrent chemoradiation therapy (CCRT) with weekly cisplatin (cisplatin 40 mg/m^2^/week for 5–6 weeks). All patients were followed every 1–3 months for 1 year after initial treatment, subsequently every 3 months for 3 years with the gynecological examination, transvaginal ultrasonography, cervical Pap smear, biopsy, serum tumor marker-level monitoring, computed tomography, and magnetic resonance imaging. The clinical response was determined using the Response Evaluation Criteria in Solid Tumors, version 1.1 [13].

### Immunohistochemical staining and pathological assessment

**Fig. 1:**
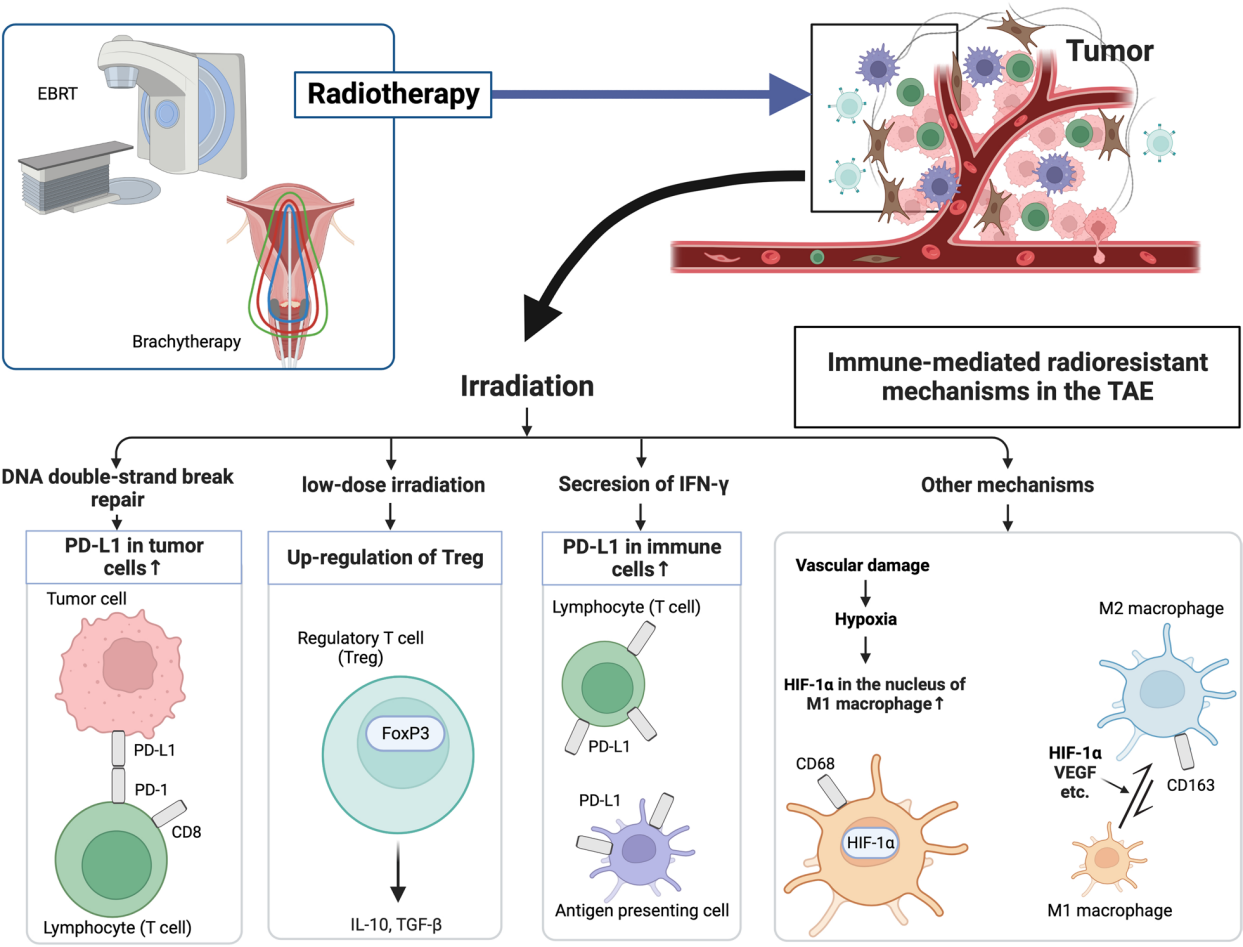
Immune-mediated radioresistant mechanisms in the tumor microenvironment and related molecules. Black arrows depict the immune responses involved in radioresistance that have been identified so far. Radiotherapy for cervical cancer involves external-beam radiation therapy (EBRT) and brachytherapy, although little is known about the effects of the different doses per fraction and irradiation schedules of these two treatment modalities on tumor immunity (blue arrow). *EBRT* external beam radiotherapy, *TME* tumor microenvironment, *DNA* deoxyribonucleic acid, *CD* cluster of differentiation, *PD-L1* programmed death ligand 1, *PD-1* programmed death 1, *FoxP3* forkhead boxprotein P3, *HIF-1α* hypoxia-inducible factor-1α, *VEGF* vascular endothelial growth factor, *IL* interleukin, *TGF-β* transforming growth factor-β

We conducted this study using all the pathological tissues stored at our institution. After each biopsy, the tissues were immediately fixed in 10% formalin and embedded in paraffin. The biopsy specimens showed that cancer cells were collected in most cases in the early stages of treatment. However, as the treatment progressed, cancer cells were not collected. It was impossible to confirm whether the treatment was successful if the cancer cells were destroyed or whether surviving cancer cells were not collected during the biopsy. Given the uncertainty that biopsies were not always guaranteed to collect tumor tissues, we decided to focus on tumor-associated immunity in the TME rather than tumor cells. To investigate changes in tumor-associated immunity in TME, we first selected samples at four-time points among the cervical tissues mentioned above: before treatment (biopsy point 1), at the midpoint of the EBRT-only irradiation period (biopsy point 2), at the time points when the HDR-ICBT dose was approximately half of the planned dose (biopsy point 3), and within 3 months of the end of treatment (biopsy point 4). Regarding the prescription dose for the external os of the uterus at each time point, biopsy points 2, 3, and 4 were EBRT 29.7 (16.0–37.8) Gy, 31.5 (19.8–50.4) Gy + HDR-ICBT 53.3 (15.2–100.5) Gy, and 31.5 (19.8–50.4) Gy + HDR-ICBT 107.1 (34.3–240.4) Gy. Subsequently, serial 4-μm sections were cut from these specimens, and immunohistochemical staining was conducted using the primary antibodies shown in Table 1. Figure 1 depicts the functions of the immune-related molecules investigated in this study within the TME [14–21]. CD8 + T cells recognized tumor antigens by the action of antigen-presenting cells. PD-1 is a receptor expressed on the surface of activated T cells. PD-L1 is a ligand for PD-1 and is also expressed on the surface of tumor cells and macrophages surrounding tumors. PD-1 and PD-L1 bind to each other, suppressing or arresting T-cell responses. CD68 and CD163 are highly expressed in macrophages, with CD68 being more prevalent in M1 macrophages and CD163 in M2 macrophages. M1 macrophages typically demonstrate anti-tumor functions, including directly mediating cytotoxicity and antibody-dependent cell-mediated cytotoxicity to kill tumor cells. However, M2 macrophages can activate the recurrence and metastasis of tumor cells, inhibit T cell-mediated anti-tumor immune response, promote tumor angiogenesis, and result in tumor progression. FoxP3 is a typical marker of regulatory T cells. Hypoxia-inducible factor-1α (HIF-1α), a transcription factor activated during intracellular hypoxia, activates M2 macrophages; it is expressed in the nuclei of M1 macrophages. Expression levels were defined as the ratio of the number of cells expressing the target molecule to the total number of cells in the TME, and they were determined by the consensus of the radiation oncologist and pathologist.

**Table 1:** Primary antibodies used for immunohistochemical staining

Clone	Antigen	Dilution	Manufacturer	Cell expression site in the TME
KP-1	CD68	1:1200	DakoCytomation, Glostrup, Denmark	Cell membrane
10D6	CD163	1:100	Leica Microsystems, Newcastle, UK	Cell membrane
EPR4877 (2)	PD-1	1:200	Abcam, Cambridge, MA, USA	Cell membrane
E1L3N	PD-L1	1:100	Cell Signaling Technology, Inc., Danvers, MA, USA	Cell membrane
4B11	CD8	1:200	Leica Microsystems, Newcastle, UK	Cell membrane
236A/E7	FoxP3	1:100	Abcam, Cambridge, MA, USA	Nucleus
54/HIF-1α	HIF-1α	1:50	BD Biosciences, New Jersey, USA	Nucleus

*TME* tumor microenvironment, *CD* cluster of differentiation, *PD-1* programmed death 1, *PD-L1* programmed death ligand 1, *FoxP3* forkhead boxprotein P3, *HIF-1α* hypoxia-inducible factor-1α

### Statistical analyses

The patients were categorized into the following two groups: the treatment failure group (n = 14), which included those who developed recurrent cancer and/or metastases within 2 years after starting treatment, and the treatment success group (n = 12), including those who did not. We compared patients’ baseline characteristics between the two groups using the Wilcoxon rank sum and Fisher’s exact tests for continuous and categorical variables, respectively. We plotted the expression levels of each immunologically related molecule expressed on the cell membrane or in the nucleus in tissues obtained at the four-time points and tested the differences in the expression levels at each time point between the two groups using a mixed linear model. Subsequently, we calculated the 2-year cumulative progression-free survival (PFS) rates using the Kaplan–Meier method and performed the univariate and multivariate Cox proportional hazards regression models to examine factors related to recurrence and/or metastasis. The EBRT and HDR-ICBT doses at the external os of the uterus and the expression levels of immune-related molecules were time-dependent factors, and we determined that verification of the proportional hazards assumption was unnecessary. The 2-year PFS rates were calculated from the day of the completion of RT to the date of the last follow-up attendance, histological or radiological evidence of any recurrence or metastasis, or death from any cause. Regarding the radiation dose to the tissues collected, the biopsy samples used in this study were collected around the external os of uterus; therefore, the dose of EBRT was calculated up to the time before the midline block was administered, and the dose of HDR-ICBT was calculated at a distance of 5 mm perpendicular to the longitudinal axis of the uterus from the external os of uterus using HDRplus™. However, due to the small number of patients in this study, the explanatory variables to be included in the multivariate analysis were age and the doses of EBRT and HDR-ICBT in the external os of uterus, which were considered to be clinically significant in addition to each level of expression of immune-related molecules. We conducted comparisons of baseline characteristics of patients between the two groups using the RStudio, version 1.3.1056 (RStudio Team (2020). RStudio: Integrated Development by R. RStudio, Inc., Boston, MA, USA), and confirmed differences in the expression levels at each time point between the two groups and performed the univariate and multivariate analyzes using the SAS version 9.4 (SAS Institute Inc., Cary, NC, USA).

## Results

### Patients’ characteristics

The clinical characteristics of the patients are presented in Table 2. The median age of the treatment success group was higher than that of the treatment failure group (*p* = 0.03). In addition, a significant difference was observed in the efficacy of CCRT between the two groups, and all patients diagnosed with partial response were in the treatment failure group (*p* = 0.02). The remaining explanatory variables and treatment effects did not differ significantly between the two groups.

**Table 2:** Baseline characteristics of patients

Characteristics	Treatment success	Treatment failure	*p-value*	Characteristics	Treatment success	Treatment failure	*p-value*
	(n = 12)	(n = 14)			(n = 12)	(n = 14)	
Age (year)				EBRT field, n (%)			0.46
Median (range)	61 (47–89)	49 (33–72)	0.03	Whole pelvis	11 (92)	14 (100)	
ECOG Performance Status, n (%)			0.78	Small pelvis	1 (8)	0 (0)	
0	10 (83)	12 (86)		Chemotherapy, n (%)			0.58
1	1 (8)	2 (14)		0 mg/m^2^	2 (17)	1 (7)	
2	1 (8)	0 (0)		≥ 100 mg/m^2^	10 (83)	13 (93)	
Histology, n (%)			1.00	EBRT dose, n (%)			0.20
Squamous cell carcinoma	12 (100)	13 (93)		1.8 Gy × 28 fr	10 (83)	14 (100)	
Adenocarcinoma	0 (0)	1 (7)		1.6 Gy × 31 fr	1 (8)	0 (0)	
Stage (FIGO 2009), n (%)			0.51	2.0 Gy × 15 fr	1 (8)	0 (0)	
I	1 (8)	3 (21)		HDR-ICBT dose at Point A, n (%)			0.59
II	6 (50)	9 (64)		5 Gy × 2 fr	1 (8)	0 (0)	
III	3 (25)	1 (7)		5 Gy × 4 fr	0 (0)	1 (7)	
IV	2 (17)	1 (7)		5 Gy × 5 fr	3 (25)	3 (21)	
Tumor diameter, n (%)			1.00	5 Gy × 6 fr	8 (67)	6 (43)	
< 4 cm	2 (17)	2 (14)		5 Gy × 7 fr	0 (0)	2 (14)	
≥ 4 cm	10 (83)	12 (86)		5 Gy × 8 fr	0 (0)	1 (7)	
Lymph node metastasis, n (%)			0.23	5 Gy × 5 fr. + 6 Gy × 1 fr	0 (0)	1 (7)	
Negative	7 (58)	4 (29)		Treatment effect, n (%)			0.02
Positive	5 (42)	10 (71)		CR	12 (100)	8 (57)	
Distant metastasis, n (%)			0.58	PR	0 (0)	6 (43)	
Negative	10 (83)	13 (93)		SD	0 (0)	0 (0)	
Positive	2 (17)	1 (7)		PD	0 (0)	0 (0)	

*ECOG* Eastern Cooperative Oncology Group, *FIGO* International Federation of Gynecology and Obstetrics, *EBRT* external-beam radiation therapy, *HDR-ICBT* high-dose-rate intracavity brachytherapy, *CR* complete response, *PR* partial response, *SD* stable disease, *PD* progressive disease

### Alteration in the expression of immune-related molecules in TME

**Fig. 2:**
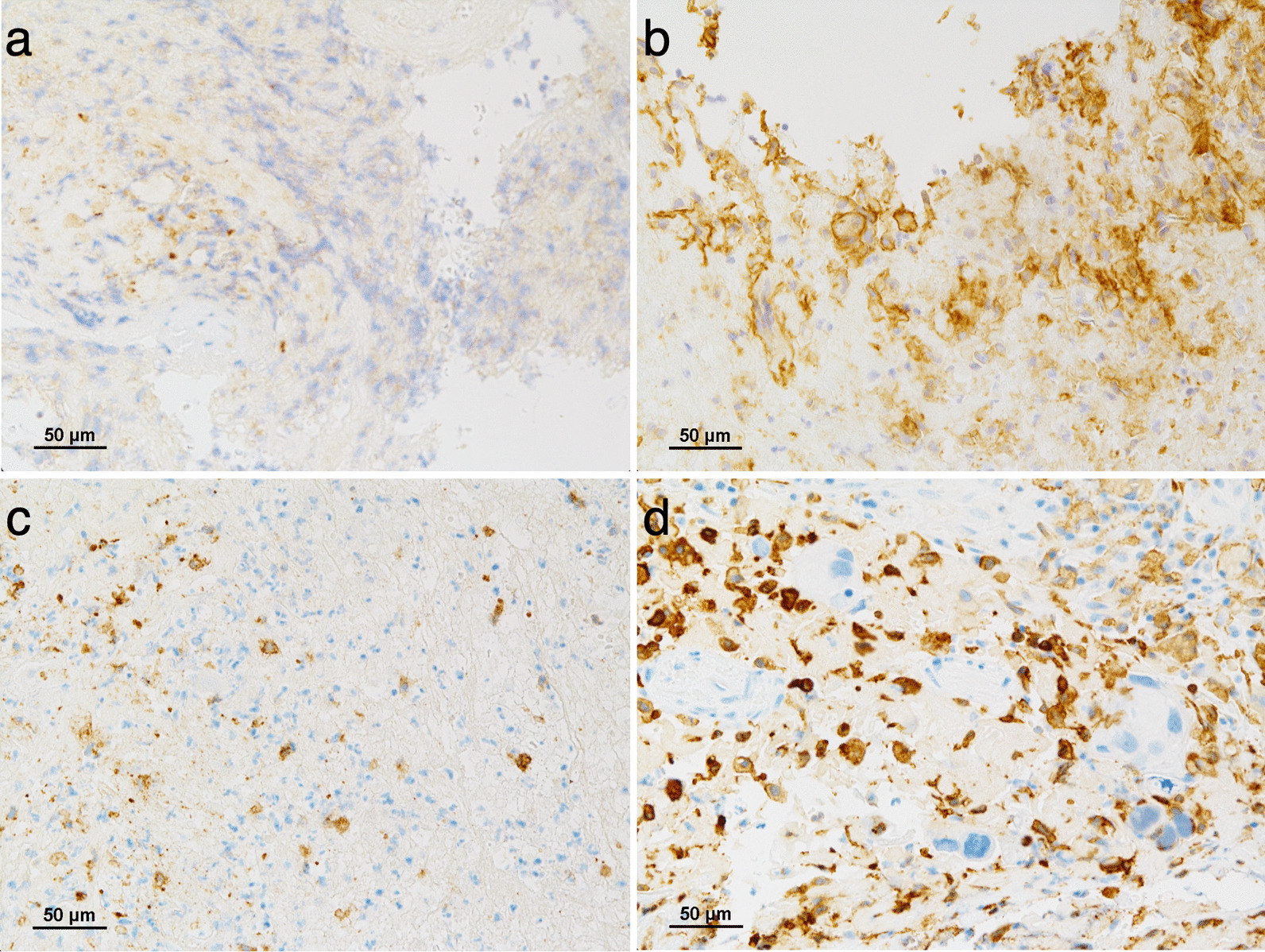
Representative patterns of immunohistochemical staining and pathological assessment of the tumor microenvironment (TME) stained for PD-L1 and CD163. Expression levels were defined as the ratio of the number of cells expressing the target molecule to the total number of cells in the TME. PD-L1 expression levels were (**a**) 5% and (**b**) 40%. CD163 expression levels were (**c**) 10% and (**d**) 50%. *PD-L1* programmed death ligand 1, *CD* cluster of differentiation

**Fig. 3:**
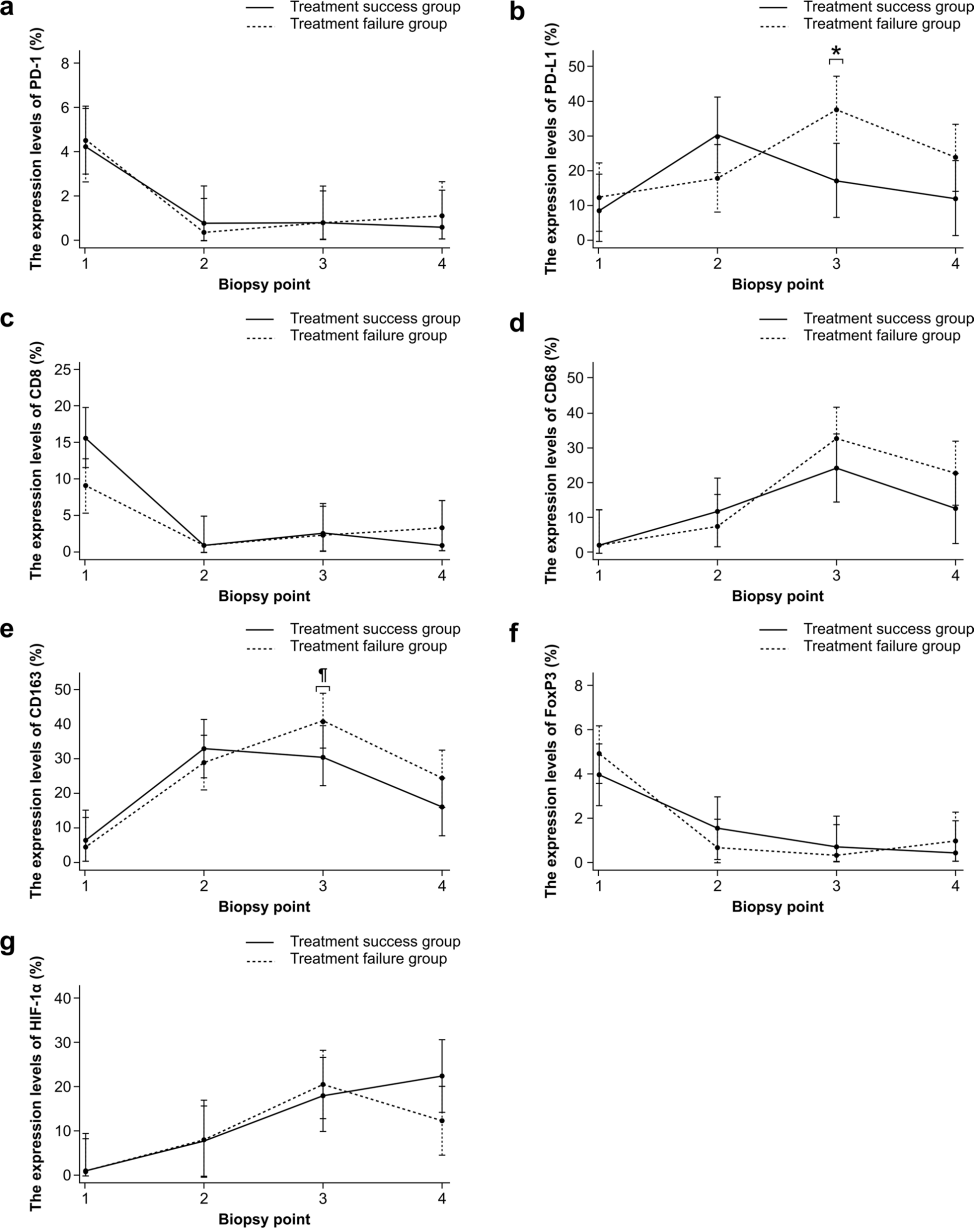
Expression levels of each immune-related molecule in the tumor microenvironment (TME) in the treatment success and treatment failure groups. **a** PD-1, **b** PD-L1, **c** CD8, **d** CD68, **e** CD163, **f** FoxP3, and **g** HIF-1α. We defined four biopsy points as follows: biopsy point 1, before treatment; biopsy point 2, at the midpoint of the EBRT-only irradiation period; biopsy point 3, at the time points when the HDR-ICBT dose was approximately half of the planned dose; biopsy point 4, after treatment. *The expression levels of PD-L1 in the treatment success group were lower than those in the treatment failure group at biopsy point 3 (*p* < 0.01). ¶The same trend was observed for CD163 at biopsy point 3 (*p* = 0.08). *PD-1* programmed death 1, *PD-L1* programmed death ligand 1, *CD* cluster of differentiation, *FoxP3* forkhead boxprotein P3, *HIF-1α* hypoxia-inducible factor-1α, *EBRT* external beam radiotherapy, *HDR-ICBT* high-dose-rate intracavity brachytherapy

As an example, representative patterns of TMEs stained for PD-L1 and CD163 are shown in Fig. 2. The expression levels of each immune-related molecule in TME in the two groups are shown in Fig. 3 and Table 3. We confirmed the differences in the expression levels at each time point between the two groups and found the following results: the expression levels of PD-L1 increased at biopsy point 2, compared to biopsy point 1, and the expression levels of PD-L1 in the treatment success group were lower than those in the treatment failure group at biopsy point 3 (*p* < 0.01). The same trend was observed for CD163 (*p* = 0.08). In addition, the expression levels of CD8, FoxP3, and PD-1 gradually decreased, and the expression levels of CD68 and HIF-1α gradually increased during treatment. However, no significant difference was found in the expression levels of these molecules at almost all of the biopsy points between the two groups.

**Table 3:** Results of the mixed linear model for each immune-related molecule

	PD-1	*p-value*	PD-L1	*p-value*	CD8	*p-value*	CD68	*p-value*	CD163	*p-value*	FoxP3	*p-value*	HIF-1α	*p-value*
*Treatment*														
Treatment success group (Comparison object)	0.0	–	0.0	–	0.0	–	0.0	–	0.0	–	0.0	–	0.0	–
Treatment failure group	0.3 ± 1.1	0.82	3.8 ± 7.3	0.61	− 6.6 ± 2.8	0.02	0.0 ± 6.9	1.00	− 1.4 ± 5.9	0.82	0.9 ± 0.8	0.34	− 0.6 ± 5.7	0.92
*Biopsy point*														
Point 1 (Comparison object)	0.0	–	0.0	–	0.0	–	0.0	–	0.0	–	0.0	–	0.0	–
Point 2	− 3.4 ± 1.1	< 0.01	21.5 ± 6.9	< 0.01	− 14.9 ± 2.9	< 0.01	9.3 ± 6.0	0.12	26.3 ± 6.0	< 0.01	− 2.4 ± 1.0	0.01	6.9 ± 6.1	0.25
Point 3	− 3.4 ± 1.1	< 0.01	8.5 ± 6.9	0.21	− 13.2 ± 2.9	< 0.01	22.0 ± 6.0	< 0.01	23.8 ± 6.0	< 0.01	− 3.3 ± 1.0	< 0.01	16.8 ± 5.9	< 0.01
Point 4	− 3.6 ± 1.1	< 0.01	3.3 ± 6.9	0.62	− 14.9 ± 2.9	< 0.01	10.4 ± 6.0	0.09	9.7 ± 6.0	0.11	− 3.5 ± 1.0	< 0.01	20.9 ± 5.9	< 0.01
*Interaction terms (treatment and biopsy point)*														
Differences between treatment groups for changes between points 1 and 2	− 0.7 ± 1.5	0.66	− 16.1 ± 9.3	0.08	6.6 ± 4.0	0.09	− 4.1 ± 8.2	0.62	− 2.9 ± 8.2	0.72	− 1.8 ± 1.3	0.18	0.2 ± 8.2	0.98
Differences between treatment groups for changes between points 1 and 3	− 0.3 ± 1.5	0.84	16.6 ± 9.2	0.07	6.4 ± 4.0	0.11	8.4 ± 8.2	0.31	11.7 ± 8.2	0.15	− 1.3 ± 1.3	0.33	2.8 ± 8.1	0.73
Differences between treatment groups for changes between points 1 and 4	0.2 ± 1.5	0.89	8.0 ± 9.2	0.39	8.9 ± 4.0	0.02	10.3 ± 8.2	0.21	9.6 ± 8.2	0.24	− 0.4 ± 1.3	0.77	− 9.3 ± 8.1	0.25
*Differences in expression levels between treatment groups at biopsy points*														
Point 3	0.0 ± 1.1	0.98	0.0 ± 1.1	< 0.01	− 0.2 ± 2.8	0.95	8.4 ± 6.9	0.22	10.4 ± 5.9	0.08	− 0.4 ± 1.0	0.71	2.2 ± 5.7	0.70
Point 4	0.5 ± 1.1	0.67	0.5 ± 1.1	0.11	2.4 ± 2.8	0.40	10.3 ± 6.9	0.14	8.2 ± 5.9	0.17	0.5 ± 1.0	0.58	− 9.9 ± 5.7	0.08

We selected samples at four-time points among the cervical tissues: before treatment (biopsy point 1), at the midpoint of only the external beam radiation therapy period (biopsy point 2), at the time points when the high-dose-rate intracavity brachytherapy dose was approximately half of the planned dose (biopsy point 3), and within three months of the end of treatment (biopsy point 4). PD-L1, programmed death ligand 1

### Prognosis

The median follow-up period was 32 (5–75) months. During this study, recurrence and/or metastasis were observed in 14 (54%) patients: local recurrence in 10 (38%), distant metastasis in seven (27%), and both local recurrence and distant metastasis in three (12%) patients. Five (19%) patients died of uterine cervical cancer. The 2-year PFS and overall survival rates in all 26 patients were 46% and 81%, respectively. The results of the univariate and multivariate analyses are presented in Tables 4 and 5. Although the univariate analysis showed no significant association between the 2-year PFS and the expression level of any immune-related molecule, the multivariate analysis revealed that the expression levels of PD-L1 (hazard ratio [HR] 1.033; 95% confidence interval [CI] 1.00–1.07*; p* = 0.04) and CD163 (HR 1.056; 95% CI 1.01–1.10*; p* = 0.02) were independently associated with the 2-year PFS, whereas those of other molecules were not significantly associated with the 2-year PFS.

**Table 4:** Predictive factors of the 2-year PFS in univariate analysis

Variables	Univariate analysis		
	HR	95% CI	*p-value*
Age	0.95	(0.91, 1.00)	0.03
FIGO stage	0.67	(0.34, 1.34)	0.26
Lymph node metastasis			
Negative	1.00	–	–
Positive	2.13	(0.65, 6.91)	0.21
Distant metastasis			
Negative	1.00	–	–
Positive	0.00	(0.00, ∞)	0.99
EBRT dose at the external os	1.04	(0.98, 1.11)	0.17
HDR-ICBT dose at the external os	1.01	(0.99, 1.03)	0.28
Chemotherapy			
0 mg/m^2^	1.00	–	–
≥ 100 mg/m^2^	1.01	(0.99, 1.02)	0.35
PD-1 expression levels	1.15	(0.72, 1.84)	0.56
PD-L1 expression levels	1.02	(1.00, 1.05)	0.09
CD8 expression levels	1.08	(0.95, 1.22)	0.24
CD68 expression levels	1.02	(0.99, 1.04)	0.26
CD163 expression levels	1.03	(1.00, 1.06)	0.09
FoxP3 expression levels	1.17	(0.65, 2.11)	0.61
HIF-1α expression levels	0.99	(0.96, 1.02)	0.52

*PFS* Progression-free survival, *FIGO* International Federation of Gynecology and Obstetrics, *EBRT* external-beam radiation therapy, *HDR-ICBT* high-dose-rate intracavity brachytherapy, *HR* hazard ratio, *CI* confidence interval

**Table 5:** Predictive factors of the 2-year PFS in multivariate analysis

Variables	Multivariate analysis		
	HR*	95% CI	*p-value*
PD-1 expression levels	1.18	(0.68, 2.05)	0.56
PD-L1 expression levels	1.03	(1.00, 1.07)	0.04
CD8 expression levels	1.13	(0.97, 1.32)	0.13
CD68 expression levels	1.03	(0.99, 1.06)	0.19
CD163 expression levels	1.06	(1.01, 1.10)	0.02
FoxP3 expression levels	1.24	(0.67, 2.30)	0.49
HIF-1α expression levels	1.00	(0.97, 1.03)	0.89

These data were adjusted for age, EBRT dose at the external os, and HDR-ICBT dose at the external os

*PFS* progression-free survival, *HR* hazard ratio, *CI* confidence interval, *PD-L1* programmed death ligand 1, *EBRT* external-beam radiation therapy, *HDR-ICBT* high-dose-rate intracavity brachytherapy

*HR was calculated as an increase in one unit of expression level

## Discussion

This study aimed to determine whether changes in the immunity of the TME during standard radical RT for cervical cancer combined with EBRT and brachytherapy affect prognosis. It revealed that the expression levels of PD-L1 and CD163 in the treatment success group were lower than those in the treatment failure group at the midpoint during brachytherapy and that the 2-year PFS rate depended on the expression levels of PD-L1 and CD163.

Several studies have demonstrated that the expression levels of PD-L1 increase following RT, which aided in the interpretation of our findings; the expression levels of PD-L1 increased before the initiation of ICBT [10, 22]. However, subsequently, the dynamics of PD-L1 expression levels differed depending on whether recurrence or metastasis occurred within 2 years. This result suggests that the incidence of recurrence and/or metastasis within 2 years depends on the expression levels of PD-L1 during brachytherapy.

Macrophages are one of the main components of tumor-infiltrating monocytes. These cells, which are involved in tumor immunity in the TME, are classified as M1 and M2 macrophages, and the former is generally considered to have an anti-tumor phenotype with cytotoxic capabilities. In contrast, the latter has a tumor-promoting phenotype with immunosuppressive and angiogenic capabilities that suppress tumor immunity [15, 17, 23, 24]. Our study showed that the expression levels of CD68 and CD163 increased with treatment progression, contrary to the findings of Berenguer et al. [22]. These differences may be because of the disuniformity in pathological evaluation arising from variations in the biopsy site, insufficient specimen volume, or the small number of cases. However, in this study, the expression levels of PD-L1, which is expressed on the surface of myeloid-derived suppressor cells: the precursor cells of M1 and M2 macrophages, and tumor-associated macrophages: a collective term for macrophages within the TME [23, 24], as well as HIF-1α, which is expressed in the nucleus of M1 macrophages and an activator of M2 macrophages [17, 21], increased during treatment. These results may support increased expression levels of CD68 and CD163.

Regarding the expression levels of PD-1, FoxP3, and CD8 in the TME, Tsuchiya et al. investigated the expression rates of immune-related molecules and demonstrated that after CCRT, CD8 + and FoxP3 + T-cell infiltration decreased significantly, while the number of PD-1-expressing cells did not change [25]. This result is consistent with our result. In contrast, Someya et al. reported that low FoxP3 + T-cell count and low CD8 + T-cell count (cold-type tumors) in both the tumor and TME before RT were poor prognostic factors, in addition to non-squamous cell carcinoma, large pretreatment tumor volume, and three or fewer cycles of concurrent chemotherapy [26]. Here, we did not identify any difference in CD8 expression before treatment according to treatment outcomes, and this is because Someya et al. distinguished infiltration by counting cells in the tissue above or below 30 cells/HPF, whereas we counted cells without dichotomization; consequently, some of the patients in the treatment success group in our study were included in their cold-type group, and the results differed regarding the expression levels of these markers before treatment.

Here, the underlying reasons for the discrepancy in PD-L1 and CD163 expression rates according to prognosis at the midpoint of brachytherapy are yet to be determined. Given that HIF-1 increases PD-L1 expression on M2 macrophages in hypoxic environments [23, 27, 28] and that the therapeutic efficacy of X-ray or γ-ray is regulated by tissue oxygenation [29], we considered hypoxia as a possible explanation for this prognostic difference. Although our study showed that the expression level of HIF-1α was unrelated to the prognosis and increased during treatment, we only examined one of several HIF proteins in this study. The possibility that a phenomenon in the hypoxic environment may have generated prognostic variations in PD-L1 and CD163 expression in brachytherapy could not be ruled out by our findings; this subject requires further research.

Cellular senescence and aging are associated with PD-L1 upregulation, and a large variety of proinflammatory cytokines, chemokines, growth factors, and proteases secreted by senescent cells upregulate PD-L1 expression in non-senescent control cells via the JAK-STAT pathway [30]. However, the median age of the treatment success group was higher than that of the treatment failure group in our study. This is possibly owing to patient selection bias because our study was a retrospective analysis of patients who completed radical treatment; therefore, patients who were unable to complete treatment or could not be treated radically may not have been included in the study.

This study had some limitations. First, the study had an exploratory and preliminary nature because of the small sample size and the uncertainty of the pathological evaluation arising from variations in the biopsy site or insufficient specimen volume. Second, when evaluating tissue samples, we did not score expression levels as other researchers have, nor did we determine the cutoff point, making the results of this study difficult to reflect in actual clinical practice. Therefore, to overcome these constraints, we intend to increase the number of patients and conduct further investigations to determine the underlying reasons for the prognosis during brachytherapy and the timing of immunotherapy initiation.

## Conclusions

This exploratory study of cervical cancer revealed that patients with no tumor progression within 2 years after starting treatment had lower expression levels of PD-L1 and CD163 at the brachytherapy midpoint and that the expression rate of these molecules was related to the 2-year PFS. Although it is necessary to investigate the underlying causes of the differences in PD-L1 and CD163 expression in the TME during brachytherapy, this study may increase our understanding of tumor-associated immunity and aid in the development of treatment methods that exploit this immunity in RT for cervical cancer.

## Data Availability

The datasets used and/or analyzed during the current study are available from the corresponding author on reasonable request.

## Abbreviations

TME: Tumor microenvironment
CD: Cluster of differentiation
PD-L1: Programmed death ligand 1
EBRT: External beam radiotherapy
HPV: Human papilloma virus
PD-1: Programmed death 1
FoxP3: Forkhead boxprotein P3
HIF-1α: Hypoxia-inducible factor-1α
RT: Radiotherapy
DSB: Double-strand break
HDR-ICBT: High-dose-rate intracavity brachytherapy
CCRT: Concurrent chemoradiation therapy
PFS: Progression-free survival
